# Leptomeningeal carcinomatosis in gastric cancer: A Review

**DOI:** 10.1007/s10120-025-01597-8

**Published:** 2025-03-14

**Authors:** Simran Arjani, Hyein Jeon, Bhawneet Chadha, Huda Yousuf, Enrico Castellucci

**Affiliations:** 1 Department of Internal Medicine, Montefiore Medical Center Albert Einstein College of Medicine, 111 East 210 Street, Bronx, NY 10467 USA; 2 Department of Oncology, Montefiore Medical Center Albert Einstein Comprehensive Cancer Center, Bronx, NY USA

**Keywords:** Leptomeningeal disease, Leptomeningeal metastasis, Gastric cancer, Gastric adenocarcinoma

## Abstract

Gastric cancer is the fifth most common cancer worldwide and leptomeningeal carcinomatosis (LM) occurs in 0.06% of gastric cancers. As such, trials are difficult to power and quantitative analyses difficult to standardize. We composed a review and analysis of 47 recent cases to be used as a comprehensive resource for an oncologist faced with managing this highly morbid, rapidly fatal disease. Gold-standard of diagnosis of LM is through cerebral spinal fluid (CSF) cytology; MRI is the preferred imaging modality to identify LM. However, repeated lumbar punctures and imaging studies are often required to establish diagnosis. Negative results do not rule out LM. Treatment includes radiation and intrathecal chemotherapy, most commonly with methotrexate. Systemic treatment with chemotherapy and immunotherapy is also used. Median survival was 2 months. Intrathecal methotrexate was most commonly dosed at 10-12 mg and treatment continued till symptom resolution, serial lumbar punctures with negative cytology, decrease and stabilization of CSF carcinoembryonic antigen (CEA) levels, progression of disease, or poor functional status. The maximum survival was 12 months. The results of this review indicate that suspicion for leptomeningeal disease should be high in any patient with gastric malignancy or with symptoms consistent with malignancy. Treatment on a biweekly to bi-monthly basis and the addition of systemic therapy to intrathecal therapy should be studied in a matched prospective manner. And in the absence of this information, treatment with at least intrathecal chemotherapy and radiation therapy should be considered in those with a performance status conducive to continued treatment.

## Introduction

**Fig. 1:**
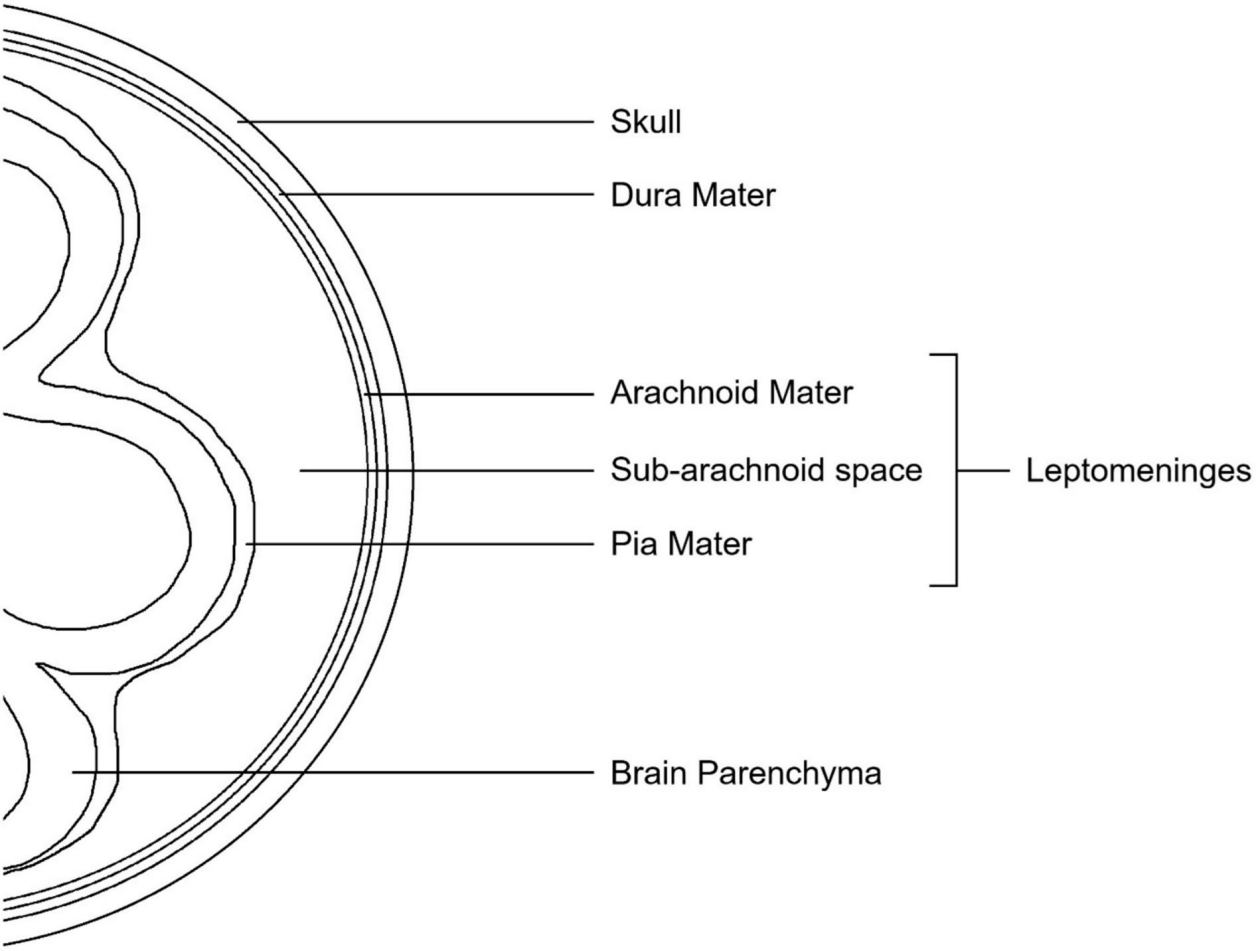
Diagram specifying the leptomeninges and what is meant by leptomeningeal involvement. Leptomeningeal carcinomatosis does not include metastases to the brain parenchyma or the dura mater, through these spaces can become invaded concordantly

Gastric cancer is the fifth most common cancer in the world [1], and 26,500 new cases are expected to be diagnosed in 2023 in the United States [2]. The estimated five-year relative survival of gastric cancer is 35.7% and at least 33.9% have distant spread at diagnosis [3]. Very rarely, those diagnosed with distant disease have leptomeningeal involvement. Leptomeningeal carcinomatosis (LM) has been reported to occur in 1 – 8% of all cancer patients [4] and in 5 – 15% of all stage IV cancers [5]; it is described as the involvement of the arachnoid mater, the pia mater, and the subarachnoid space (Fig. 1). It most commonly occurs in breast, lung, and melanoma cancers as well as in primary CNS cancers, non-Hodgkin lymphoma, acute lymphoblastic leukemia, and multiple myeloma [4]. In gastric cancer it is exceedingly rare. Its incidence is not commonly reported as most of the published literature on this topic is composed of case reports and case series. A study from Korea published in 1999 that analyzed 8080 patients with advanced gastric carcinoma found that five patients, representing 0.06% of the patient population, had LM [6].

Because of the rarity of this presentation identifying the appropriate treatments for these individuals is difficult, and clinical trials are very difficult to power. Multiple trials addressing LM have been proposed and then closed due to the inability to recruit enough participants [5]. Quantitative retrospective analyses of different treatment regimens are also difficult to conduct as patients do not receive standardized treatment regimens and many patients transition to palliative care soon after presentation or diagnosis with leptomeningeal involvement. In this context we have composed a review and analysis of recent cases of gastric cancer with leptomeningeal carcinomatosis. The purpose of this study is to create a comprehensive resource for an oncologist faced with managing this highly morbid, rapidly fatal disease.

## Methods

A comprehensive review of the literature was performed to analyze the presentations and treatments of leptomeningeal involvement of gastric cancer. The database PubMed was queried using the search terms “gastric” AND “adenocarcinoma” AND (“leptomeningeal” OR “meningeal”), which yielded 73 results out of which 57 discussed leptomeningeal involvement in gastric cancer. Of those articles, 22 were published in the last 10 years and were included in this review. The references of these articles were additionally reviewed for other cases within the preset time frame, bringing the total number of papers included to 25 and the total numbers of cases reported within these papers to 34. An additional query of “gastric” AND “leptomeningeal” AND “cancer” yielded 35 results of which 31 were relevant to this study, and 11 contained cases of gastric cancer leptomeningiosis not previously captured. Cases of pure dural metastases without leptomeningeal disease were not included because their associated survival is considered to be better than that of LM [7]. This brought the total to 36 studies and 47 cases (Table 1). These patients, their presenting symptoms, diagnostic criteria, attempted treatments, and survivals are discussed here.

**Table 1:** Patient demographics

Characteristics	Value (n = 47)
Age, years	
Mean	53
Range	22 – 84
Sex, % (n)	
Male	59.6 (28)
Race, % (n)	
Hispanic	6.4 (3)
Asian	27.7 (13)
Other	2.1 (1)
Not reported	63.8 (30)

*n* number

Clinical manifestations of LM were classified into three categories based on which portion of the nervous system was involved: cerebral signs, cranial nerve signs, or spinal signs. The use of computed tomography (CT), magnetic resonance imaging (MRI), and positron-emission tomography (PET) as well as their ability to identify LM were reported. Lumber puncture (LP) results included opening pressure, cell count, glucose, and protein levels. Normal opening pressure for an adult was an upper limit of 250 mmH2O. Some sources recommend that the upper limit be 200 mmH2O but in patients with a higher BMI a higher pressure can be tolerated. In the setting of this ambiguity, a cutoff of 250 mmH2O was used to classify patients as having elevated opening pressures [8]. A cutoff of five cells or fewer was considered normal for white blood cells (WBC), and if red blood cell (RBC) counts were recorded, one WBC was subtracted for every 500 RBCs [8]. Glucose was considered low if the CSF-to-serum glucose ratio was < 0.5 [9] or if, in the absence of a reported serum glucose, the CSF glucose was < 35 [10]. The upper limit of normal for CSF protein was 58 mg/dL and if RBCs were reported, 1 mg/dL was subtracted for each 1000 RBCs [8]. Kaplan Meier survival curves were computed in or order to further characterize the impact of treatment on survival. Survival from time of presentation to death was used when possible. If unavailable, survival from time of symptoms onset (*n* = 4), or survival from presentation to transition to palliative care (*n* = 1) were used. Statistical analyses were performed using R programming.

## Results

### Presenting signs and symptoms

There were 47 cases identified, ranging in age from 22 – 84 years. The mean age was 53 years (95% CI 49.2 – 57.2y); 40.4% of patients were female (Table 1). All 47 cases presented with some clinical evidence of leptomeningeal involvement (Table 2). Cerebral signs or signs of increased intracranial pressure were present in 89.4% of people. These included headache, nausea or vomiting, blurred vision, neck stiffness, generalized seizure, altered mentation, confusion, ataxia, dysphasia, dysgraphia, and loss of consciousness. Cranial nerve deficits were present in approximately 51.1% of cases and included ptosis, dizziness, vertigo, ear fullness, hearing loss, and dysarthria. Spinal signs were the least common at 23.4% and presented as extremity pain, numbness, weakness, low back pain, or gait difficulty. Two cases showed non-specific generalized symptoms of fever, fatigue, or night sweats. In all cases, spinal signs occurred with concurrent cerebral or cranial nerve signs and never in isolation. Due to rapid progression of disease, over days to weeks, many people developed signs in multiple categories (53.2%).

**Table 2:** Clinical signs and symptoms at initial presentation

Clinical Feature	Frequency (n)	Percentage (%)
Headache	28	59.6
Nausea/vomiting	13	27.7
Vision changes	10	21.3
Dizziness	9	19.1
Abdominal pain	5	10.6
Altered mental status	5	10.6
Weakness	5	10.6
Dyspepsia	2	4.3
Seizure	2	4.3
Loss of consciousness	1	2.1
Incidental	1	2.1

*n* number

### Imaging

The initial diagnosis of leptomeningeal metastases (LM) often relies heavily on imaging studies, particularly in the context of nonspecific symptoms and signs at presentation. Patients frequently undergo an infectious workup and brain imaging, especially in cases of increased intracranial pressure, to rule out bleed or mass effect. (Table 3). Most with focal neurological deficits received imaging of both the brain and the spine.

**Table 3:** Summary of diagnostic evaluations

Reference	Year	Age	Sex	CSF	MRI Imaging	Gastric Tumor/ Metastasis Pathology
Lai SY	2023	64	F	IOP, C + w/ atypical epithelioid cells	Abnormal enhancement, bilateral nerve sheathes	PDA
Shwarzova K	2023	53	M	C +	Meningeal enhancement, occipital lobe	PDA
Baleiras MM	2022	46	M	C + (SR)	LM enhancement, malignant infiltrates in cerebrum	ADA, SR
Baleiras MM	2022	50	M	C + (SR)	Hypersignal and locoregional thickening of the superior frontal sulcus suggestive of malignancy	ADA, SR
Falk Z	2022	50	F	NR	Asymmetric enhancement of superior margin of left cavernous sinus, right Meckel's cave, bilateral pre-pontine cistern, bilateral cranial nerve 6, cerebellar lesions, left occipital bone lytic lesion, CSF seeding on upper spinal cord, cervical spine LM nodules, thoracic spinal cord, cauda equina, thecal sac involvement	PDA
Moldovan TR	2022	45	F	C + (SR)	Normal	PDA, SR
Yu Z	2022	49	M	C + (ADA)	Enhanced nodules in R parietal lobe and small patchy intensities in cerebellar parenchyma	MDA
Cho JH	2021	59	M	C +	Intense sulcal enhancement and internal auditory canal enhancement in bilateral hemisphere	PDA
Geramizadeh B	2021	52	M	C + (SR), ↑ protein, ↓ glucose, ↑ LDH ^a^	Increased signal intensity in subarachnoid space and sulci of both occipital lobes	ADA, SR
Ino R	2021	77	F	C- × 3, ↑ protein, ↓ glucose, 9 mono-, 4 poly-nuclear cells/uL, CEA 117.1 ng/ML	Diffuse LM enhancement of cortical surface of cerebrum, cerebellar folia, ventral surface of brainstem; linear and nodular enhancement surface of spinal cord and cauda equine	PDA
Roohe SL	2021	67	M	C + (large cell carcinoma)	Initial: Left eye swelling of rectus lateralis muscle Repeat: LM at cranial nerve VII and VIII bilaterally	MDA
Cioni E	2020	39	F	C + (SR)	Dural parafalcine nodular lesions, diffuse bihemispheric dural neoplastic involvement without LM	ADA, SR
Patan S	2020	32	F	C + (ADA)	New pachymeningeal (dura-arachnoid) enhancement, infiltrates throughout cervical, thoracic, and lumbar spine	ADA, SR
Cunha R	2019	51	M	C-	Initial: expansive lesion in the left cerebellum Repeat: multiple nodular lesions in the subarachnoid space along the cerebellum, medulla, thecal sac, compatible with LM	ADA
Kano M	2019	48	M	IOP	LM gadolinium enhancement in subarachnoid pattern	ADA
Murakami Y	2019	65	M	NR	Multiple tumors in bilateral cerebellar hemispheres, LM through cerebellar folia	ADA
Jiao XD	2018	39	M	C + (ADA)	Vermis cerebelli meningeal metastasis, edema in the vermis cerebelli	PDA, SR
Kim H	2018	43	F	C + (ADA)	Initial: enhancing dural mass at lumbosacral junction, invasion right L5, S1 roots Repeat: enhancing dural lesion at left cerebellopontine angle with extension to trigeminal nerve and internal auditory canal Repeat: multiple enhancing lesions at cerebral and cerebellar hemisphere enhancing lesions at spinal cord T1/2 and T10/11	PDA
Kontourakis P	2018	57	M	C + (gastric cancer features), flow non- hematopoietic cell population	LM enhancement	ADA, SR
Kontourakis P	2018	64	F	C +	Diffuse meningeal enhancement extended into internal auditory canal and optic nerve sheaths right more than left	PDA
Kurebayashi M	2018	78	F	C + (ADA)	High-intensity around optic nerve	PDA
Ali S	2017	56	F	C +	Initial: Right PICA infarction with communicating hydrocephalus Repeat: supratentorial, infratentorial, and spinal LM enhancement	ADA, SR
Liu Y	2017	50	M	IOP, C + (ADA)	Normal	PDA with partial mucinous ADA
Vergoulidou M	2017	48	F	C + (SR)	LM enhancement, malignant infiltrates at frontal lobe and cerebellum	ADA
Asterita R	2016	64	M	C + (ADA)	Signal hyperintensity in the subarachnoid spaces	ADA, SR
Asterita R	2016	45	M	C + (SR ADA)	Pachymeningeal and LM enhancement	ADA, SR
Defaee A	2016	57	M	C + (SR), ↑ protein	Hyperintensity in several cranial nerves consistent with LM	ADA, SR
Ho TH	2016	28	F	IOP, C + (ADA), ↑ protein,	LM enhancement	PDA, SR
Ji JG	2016	84	F	C + (ADA), ↑ protein	Diffuse contrast enhancement of the meninges 1 cm focal nodule in lower right tentorium	ADA, SR
Yamasaki T	2016	35	M	C + (gastric ADA)	Initial: normal Repeat: findings consistent with leukoencephalopathy	ADA
Riesgo VJ	2015	27	F	IOP, C-	Initial: focal meningeal enhancement adjacent to left temporal region adjacent to planum sphenoidale and right anterior clinoidal region. Questionable enhancement of intraorbital optic nerve sheaths Repeat: significantly worse nodular meningeal enhancement and worse enhancing mass lesion in sphenoid sinus extending through skull base and into sella turcica, cavernous sinuses, along planum sphenoidale	ADA
Taghizadeh-Kermani A	2015	56	M	C + , ↑ protein	Normal	Adenocarcinoma
Guo JW	2014	40	F	IOP, C + (SR)	Initial: Hydrocephalus and parenchymal swelling Repeat: progressive linear and nodular enhancement along the ventral surface of the brainstem, cerebellum and C1 – T4 spinal cord, expansion of the lateral, third, and fourth ventricles concerning for communicating hydrocephalus	ADA, SR
Kawasaki A	2014	80	F	C + (ADA)	Initial: multiple acute cerebral infarctions Repeat: hydrocephalus, high signal intensities in right cerebellar hemisphere, corona radiata, caudate nucleus, left parietal lobe	ADA, SR
Kim C	2014	56	M	NR	Enhancing lesions in right internal auditory canal, right jugular fossa, bilateral cerebellomedullary cistern, multifocal small enhancing nodules consistent with LM	ADA
Kim N	2014	37	M	C +	No MRI. CT: sulcus enhancement	ADA, SR
Kim N	2014	67	M	C +	Normal	MDA
Kim N	2014	47	M	C +	Diffuse brain and spinal cord LM	Undifferentiated ADA
Kim N	2014	64	F	C +	No MRI. CT: cerebral LM	MDA
Kim N	2014	65	M	C +	No MRI. CT: cerebral LM	MDA
Kim N	2014	72	F	C +	No MRI. CT: cerebral LM	PDA, SR
Kim N	2014	42	M	C +	Sulcus enhancement	PDA
Kim N	2014	53	M	C +	Sulcus enhancement	PDA
Kim N	2014	51	M	C +	Ventricle enhancement	PDA
Kim S	2014	58	M	IOP, C-	Initial: suspected bilateral vestibular schwannoma Repeat: increased size bilateral vestibular lesions and new lesions in cisternal segments, Meckel's caves, left cerebellum, and multiple hyperintense lesions at L1 – L4 level	ADA, SR
Kon T	2014	22	M	C +	Initial: mild dilatation ventricular system, diffuse enhancement subarachnoid space Repeat: edematous lesions in cerebral cortex, subcortical white matter of cerebrum	PDA, SR
Saad N	2014	81	F	C + (SR ADA)	Normal	ADA, SR

*IOP* Increased opening pressure, *C* + Positive for malignant cells by cytology, *C-* Negative for malignant cells by cytology, *CSF* Cerebrospinal fluid, *SR* Signet ring features, *NR* Not reported, *PDA* Poorly differentiated adenocarcinoma, *M* Male, *F* Female, *MDA* Moderately differentiated adenocarcinoma, *LM* Leptomeningeal, *ADA* Adenocarcinoma, *PICA* posterior inferior cerebellar artery, *CT* Computed tomography scan, *MRI* Magnetic resonance imaging

aPost-mortem study of the CSF

Literature has shown LM is most often characterized by pial enhancement and nodularity, usually located over but not limited to the cerebral cortical surface [11]. Spinal cord imaging also frequently shows patchy nerve root involvement, sometimes accompanied by intradural nodules, particularly in the cauda equina region [12]. Most cases reviewed for this study exhibited enhancements and nodularity (Table 3). However, these imaging features are not exclusive to LM and can also occur in conditions such as meningitis, hydrocephalus or lumbar puncture-associated intracranial hypotension [13]. As such, detailed clinical context is critical in interpreting these findings.

During initial evaluation, 23 out of 47 (48.9%) cases received a CT head, 19 (40.4%) received a CT chest, CT abdomen, CT pelvis, or CT spine, seven (14.9%) received a PET/CT or gallium scan, 40 (85.1%) received an MRI brain, and four (8.5%) received an MRI spine. Of the seven patients who did not receive an MRI brain, four of the patients already had leptomeningeal disease diagnosed from CT imaging.

Evidence of LM was seen on 17.4% (4/23) of CT head images and 52.5% (21/40) of MRI brain images. Most CTs (65.2%, 15/23) and some MRIs (20.0%, 8/40) were negative for any intracranial pathology. Spine imaging was performed in 20 patients; four of these patients had symptoms that raised suspicion for spinal involvement. Of all the spinal images, only three MRI spine images demonstrated LM; two out of these three patients had spinal symptoms. PET/CT imaging or gallium scans were performed in seven patients; abnormalities were found in six patients but none diagnosed LM.

Follow up imaging included 16 repeat MRIs of the brain of which eight showed disease that was not previously demonstrated, five re-characterized known LM, and three remained negative for LM. Repeat imaging raised the sensitivity of MRI brain studies from 52.5% to 61.7%. Of the three repeat MRI spine images, all showed LM – one showed known disease and two showed new disease. CT scans of the brain were only repeated in patients with MRI-confirmed LM to evaluate extent of disease or in the setting of changing mental status, however, none showed LM.

### Lumbar punctures

Although the gold standard for diagnosing leptomeningeal carcinomatosis is by CSF cytology on lumbar puncture, the CSF may be negative in approximately 10% of patients with LM [14]. Generally, the first lumbar puncture is 50 – 60% sensitive, and repeating lumbar punctures can increase the test sensitivity to 80% [15]. Protein level above 45 mg/dL can be seen in a majority of patients with LM, and a CSF pressure > 150 mm can also be seen though these values are not specific [16].

LPs were performed and reported in 44 of the 47 cases. Measures included opening pressures, cell counts, glucose, protein, infectious work up, autoimmune markers, tumor markers, and paraneoplastic panels are described. If the LP was negative but the clinical suspicion was high for LM, it was often repeated. Serial LPs were also performed in response to high opening pressures, and to monitor response to therapy [17, 18]. Opening pressures ranged from 27mmH2O to greater than 600mmH2O with elevated opening pressures in 12/16 cases (75.0%) and an average opening pressure of 294.5 mmH2O. The average was also an underestimate as many reported opening pressures exceeded the upper limits of their assays. 7/11 cases had elevated WBCs (63.6%), and 13/17 (76.5%) had elevated protein levels. Of the 11 cases that reported CSF protein levels and cell counts, true protein-cell count dissociation only occurred in four cases. CSF glucose levels were low in 8/13 cases (61.5%). All infectious, autoimmune, and paraneoplastic workups were negative. Cytology was positive for malignant cells in 34 out of 44 initial tests (77.8%), and in those that repeated LPs three out of four showed malignant cells on repeat cytology. All cases with positive cytology were identified as having gastric cancer origin through either signet ring cell morphology or through immunostaining consistent with their known gastric cancer.

### Treatments

Of the total 47 cases, we describe treatment regimens used in 43 cases as four patients died before receiving any treatment (Table 4). Among those patients, 18.6% received systemic chemotherapy (8/43), 39.5% received intrathecal chemotherapy (17/43), 27.9% received radiation (12/43), and 39.5% received supportive care alone (17/43). 15 patients received only supportive treatments including anti-epileptics, antibiotics, systemic steroids, medications for intracranial pressure, and palliative medications for pain and anxiety at end of life. Of the remaining 28 patients, 28.6% received systemic chemotherapy, 60.7% received intrathecal chemotherapy, 42.9% underwent radiation therapy, and 14.3% underwent surgical intervention.

**Table 4:** Case treatment modalities listed in descending order of survival. When available, survival from time of diagnosis was used. If not available then time from symptom onset was used. And in the absence of both, survival from presentation or diagnosis to time when transitioned to hospice was used. Age is listed in years, survival is listed in months

Reference	Year	Age	Sex	Survival	Treatment	Treatment details
Yamasaki T	2016	35	M	12	itCTX sCTX	MTX (10 mg), cytarabine (20 mg), prednisolone (20 mg), 2x/week for 2 weeks, then weekly for 12 months S1/cisplatin, 11 months later when disease recurred irinotecan (100 mg/m^**2**^**)** IV, weekly
Liu Y	2017	50	M	11^a^	itCTX sCTX RT BSC	MTX (5 mg), 2x/week until no malignant cells on cerebrospinal fluid for three consecutive exams Oral temozolomide (75 mg/m^2^), daily till the end of RT. After RT, mFOLFOX for 6cycles Whole brain radiotherapy (30 Gy in 10 fractions) Mannitol & dexamethasone
Kim H	2018	43	F	10	itCTX RT	MTX First to lumbosacral junction (36 Gy in 12 fractions). Later to dural mass at L skull base (40 Gy in 10 fractions). Later whole brain radiotherapy & RT to intramedullary lesion at T1/T2
Kountourakis P	2017	51	M	10	itCTX	MTX (12.5 mg), 2x/week for 3 weeks, then weekly. Treatment changed for deteriorating functional status: MTX, Cytosine Arabinoside (40 mg) and Dexamethasone (4 mg), 3 times in October & 5 times in December
Kim H	2014	47	M	10	sCTX RT	
Kim H	2014	51	M	9	itCTX	MTX
Lai S	2023	64	F	7	itCTX sCTX BSC	MTX (12 mg) & hydrocortisone (25 mg), weekly for 3 months, then every 2 weeks 5-fluorouracil, leucovorin, irinotecan (FOLFIRI) Oral dexamethasone (4 mg), daily
Moldovan TR	2022	45	F	7	itCTX	MTX (12 mg), every 2 days for 6 doses, then weekly for 21 doses & docetaxel (60 mg/m^2^**)**, every 3 weeks
Jiao XD	2018	39	M	5	itCTX sCTX BSC	MTX & dexamethasone, 4 doses in 40 days S1 & trastuzumab, then treatment changed to capecitabine, trastuzumab, lapatinib Mannitol, dexamethasone
Kim H	2014	72	F	4	RT	
Kim H	2014	37	M	4	BSC	
Saad N	2014	81	F	3.5	BSC	Cerebrospinal fluid drainage with lumbar puncture for intracranial pressure
Kim H	2014	64	F	3	RT	
Risego VJ	2015	27	F	2.83	sCTX BSC	FOLFOX Patient received steroids prior to diagnosis as empiric treatment for an alternative diagnosis
Baleiras MM	2022	50	M	2.75	itCTX BSC	MTX (12 mg) & dexamethasone (4 mg), weekly for 2 weeks, then switched to supportive care
Roohe SL	2021	67	M	2	BSC	
Kano M	2019	48	M	2	itCTX Surgery BSC	MTX (15 mg) and cytarabine (40 mg) twice VP shunt Systemic steroids, antiepileptic medications
Vergoulidou M	2017	48	F	2	itCTX sCTX BSC	MTX weekly for 4 weeks 5-fluorouracil, folic acid, oxaliplatin, docetaxel (FLOT)
Kim H	2014	67	M	2	RT	
Kim C	2014	56	M	2	sCTX RT	Irinotecan Whole brain radiotherapy
Kim H	2014	53	M	2	itCTX	MTX
Guo JW	2014	40	F	2	BSC	20% mannitol, antiepileptic drugs, diuretics
Kon T	2014	22	M	2	Surgery BSC	Extraventricular drain Steroid pulse therapy
Defaee A	2016	57	M	1.6	RT BSC	Cranial RT (5 Gy in 4 fractions) High dose dexamethasone
Cunha R	2019	52	F	1.5	Surgery BSC	Patient had VP shunt prior to diagnosis Steroids, pain control, anti-anxiety medications, physical therapy
Ji JG	2016	84	F	1.5	BSC	Antiepileptic drugs
Ino R	2021	77	F	1	none	
Geramizadeh B	2021	52	M	1	none	
Patan S	2020	32	F	1	itCTX RT	MTX Spine RT
Kurebayashi M	2018	78	F	1	BSC	Steroid pulse therapy
Asterita R	2016	45	M	1	RT	External beam radiation
Kim H	2014	65	M	1	BSC	
Kim H	2014		M	1	BSC	
Kawasaki A	2014	80	F	1	none	
Asterita R	2016	64	M	0.75	itCTX	
Kim SJ	2014	58	M	0.75	RT	Whole brain radiotherapy (30 Gy in 10 fractions)
Cioni E	2020	39	F	0.5	BSC	Antiepileptic drugs, dexamethasone, mannitol
Murakami Y	2019	65	M	0.5	BSC	
Ho TH	2016	28	F	0.5	BSC	Antiepileptic drugs, dexamethasone, glycerol
Kountourakis P	2018	64	F	0.25	itCTX BSC	MTX (12.5 mg) Steroids
Yu Z	2022	49	M	0.1	none	
Taghizadeh A	2015	56	M	0.1	none	
Falk Z	2022	50	F	0	RT	Whole brain radiotherapy & spinal RT
Schwarzova K	2023	53	M	NR	itCTX	MTX for 2 months
Baleiras MM	2022	46	M	NR	BSC	Dexamethasone (16 mg/day)
Cho JH	2021	59	M	NR	itCTX Surgery	MTX Ommaya reservoir for itCTX
Ali S	2017	56	F	NR	Surgery	Lateral vertriculostomy, then VP shunt

*M* Male, *F* Female, *itCTX* Intrathecal chemotherapy, *sCTX* Systemic chemotherapy, *RT* Radiation therapy, *BSC* Best supportive care, *MTX* Methotrexate, *S1* Tegafur/gimeracil/oteracil, *VP shunt* Ventriculoperitoneal shunt,

apatient died from a traffic accident

Systemic therapy regimens included 5-fluorouricil based regimens with oxaliplatin, docetaxel, irinotecan (FLO, FOLFOX, FOLFIRI), S1 (tegafur/gimeracil/oteracil), cisplatin, paclitaxel, capecitabine, irinotecan, oral temozolomide, trastuzumab, and lapatinib. Intrathecal chemotherapy was delivered in 17 cases and included methotrexate in 16; details were not provided in one case. Of those who received intrathecal chemotherapy, 5 of 17 patients also received systemic chemotherapy as part of their treatment. Patients received intrathecal methotrexate alone in 11 cases, once with the use of an Ommaya reservoir [19]. Methotrexate dosing was disclosed in eight cases and ranged from 5 to 15 mg, with a mode of 12 – 12.5 mg in four cases. Three patients received additional cytarabine with 40 mg doses in two patients, and a 20 mg dose in the third. The frequency of intrathecal chemotherapy was reported in eight cases and was at most every two days for a limited period, and most commonly once or twice per week. Liu et al. continued twice per week dosing until lumbar punctures were free of cancer cells on three consecutive exams[20]. Yamasaki et al. slowly tapered the frequency of intrathecal dosing and continued the treatment on an outpatient basis [21]. Jiao et al., Moldovan et al., and Kountourakis et al. additionally reported continuation of intrathecal methotrexate on a weekly or biweekly basis until progression of disease or decrease in functional status [18, 22, 23]. Of the 12 patients who received radiation therapy, no details were provided in four cases, and one reported external beam radiation, and one reported cranial radiation. Of the six other patients, 50% (n = 3) received whole brain radiation therapy alone, 16.7% (n = 1) received spinal radiation alone, and 33.3% (n = 2) received both. Brain radiation doses were reported in four cases and ranged from 5 Gy in 4 fractions to 30 Gy in 10 fractions.

The acute danger in leptomeningeal disease is due to the rapid rise in intracranial pressure, which can cause seizures, shifting of brain parenchyma, and herniation. Some patients received treatment to reduced intracranial pressure (41.9%, 18/43), of which three patients received surgeries (Table 4). Medications utilized included steroids, hypertonic saline, mannitol, and glycerol. Procedures to address increased pressure included serial lumbar punctures with drainage, spinal drainage catheters, external ventricular drains, and ventriculoperitoneal shunts.

### Survival

Survival was reported in 43 cases but with inconsistent definitions. Most reported it as survival since diagnosis with LM, some as survival from symptom onset, and a few as survival from presentation to the decision to opt for full comfort care. Median survival from presentation was 2.0 months (average 3.2 months, 95% confidence interval (CI) 2.1 – 4.4 months, n = 38), and from symptom onset was 2.5 months (average 3.2 months, 95% CI 2.0 – 4.3 months, n = 17). The maximum reported overall survival was 12 months.

Of the nine patients that survived for five months or more, the treatment was not specified for two patients. One patient received some unspecified form of systemic chemotherapy and radiation therapy, while the other received intrathecal methotrexate alone (Table 4). Of the other seven cases, six specified continuous intrathecal methotrexate at doses of 10 – 12 mg per dose that continued until symptom resolution, serial lumbar punctures with negative cytology, decrease and stabilization of CSF carcinoembryonic antigen (CEA) levels, progression of disease, or poor functional status. Four out of the seven cases also received systemic chemotherapy, two received whole brain radiotherapy and one received radiation therapy to the spine, and five cases received some steroid, either intrathecal or systemic.

**Fig. 2:**
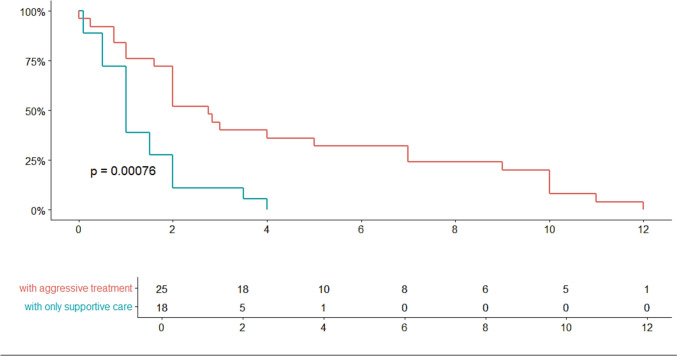
Kaplan Meier survival curve comparing those who received treatment (systemic chemotherapy, intrathecal chemotherapy, or radiation) and those who only received supportive care (anti-epileptic drugs, steroids, mannitol, etc.). Does not take into account severity of illness or patient performance status at presentation. Percent of population surviving on y-axis, time in months on the x-axis. Included table describes the number of patients surviving every 2 months

**Fig. 3:**
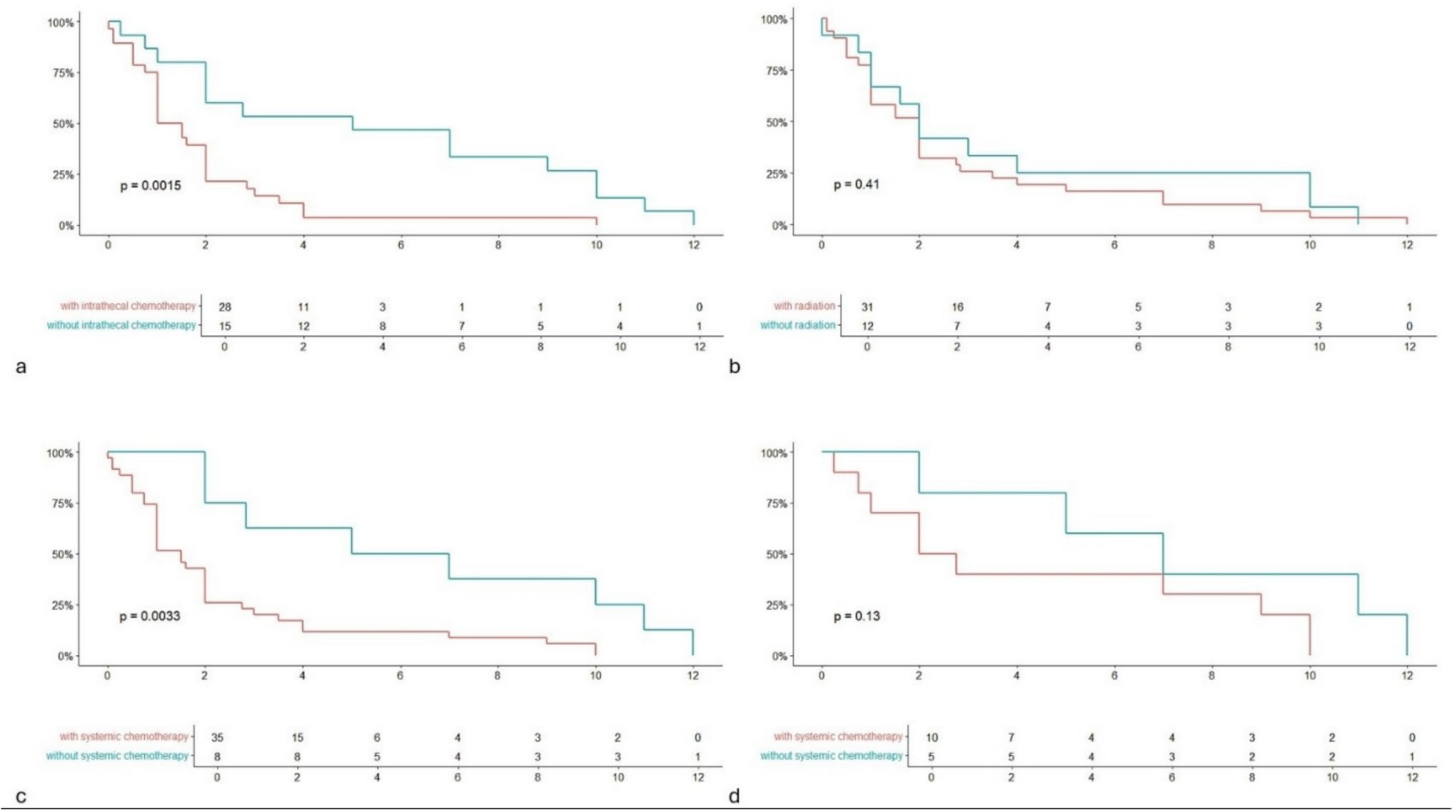
Kaplan Meier survival curves comparing survival in those who received or did not receive specific treatments: intrathecal chemotherapy (a), radiation (b), systemic chemotherapy (c). Figure 3d compares those who did and did not receive systemic chemotherapy in those who received intrathecal chemotherapy. Percent of population surviving on y-axis, time in months on the x-axis. The included tables describe the number of surviving patients at two month-intervals. Statistical significance between the two survival curves is denoted by a p value; p vale < 0.05 indicates statistical significance

By Kaplan–Meier methods the median overall survival (mOS) in months was 2.0 (95% CI 1.0 – 2. 8). A comparison of those who received treatment (systemic chemotherapy, intrathecal chemotherapy, and/or radiation) and those who only received supportive care showed a significantly increased survival in those who received aggressive treatment (Fig. 2, mOS 2.75 (2.0 – 7.0) vs 1.0 (1.0 – 2.0), *p* < 0.05). Improved survival correlated with undergoing intrathecal chemotherapy (Fig. 3a, mOS 5.0 (2.0 – 10.0) vs 1.3 (1.0 – 1.2), p < 0.05) and systemic chemotherapy (Fig. 3c, mOS 6.0 (2.8 – NA) vs 1.5 (1.0 – 2.0) (*p* < 0.05), but not with radiation (Fig. 3b, mOS 2.0 (1.0 – NA) vs 2.0 (1.0 – 2.8), *p* = 0.41). A subset analysis comparing those who did and did not receive systemic chemotherapy in those who received intrathecal chemotherapy showed no significant difference in survival between the two groups (Fig. 3d, mOS 7.0 (5 – NA) vs 2.4 (1 – NA), *p* = 0.13).

## Discussion

As cancer survival improves with the creation of new treatment regimens, the prevalence of LM is likely to increase[14]. While LM is rare, it should be considered in all patients with a cancer history and unexplained neurological symptoms. According to the Response Assessment in Neuro-Oncology (RANO) group with expertise in LM, the recommendation is to evaluate patients with a CSF profile, CSF cytology, and high quality, gadolinium enhanced MRI [14]. Negative MRI brain imaging and a lack of malignant cells in CSF also does not exclude LM. In five of the cases reviewed in this paper, CSF cytology was either negative or not obtained. In the case report by Falk, gadolinium enhanced MRI of the brain and spine demonstrated multiple scattered leptomeningeal nodules and enhancing lesions concerning for LM. Given the complexity of the patient's clinical status and the high pre-test probability for LM given the gastric adenocarcinoma with carcinomatosis, LP was not pursued [24]. Although it can be argued whether imaging alone may not be the gold standard for diagnosis for LM, on a case-specific basis, this may be a reasonable approach.

**Fig. 4:**
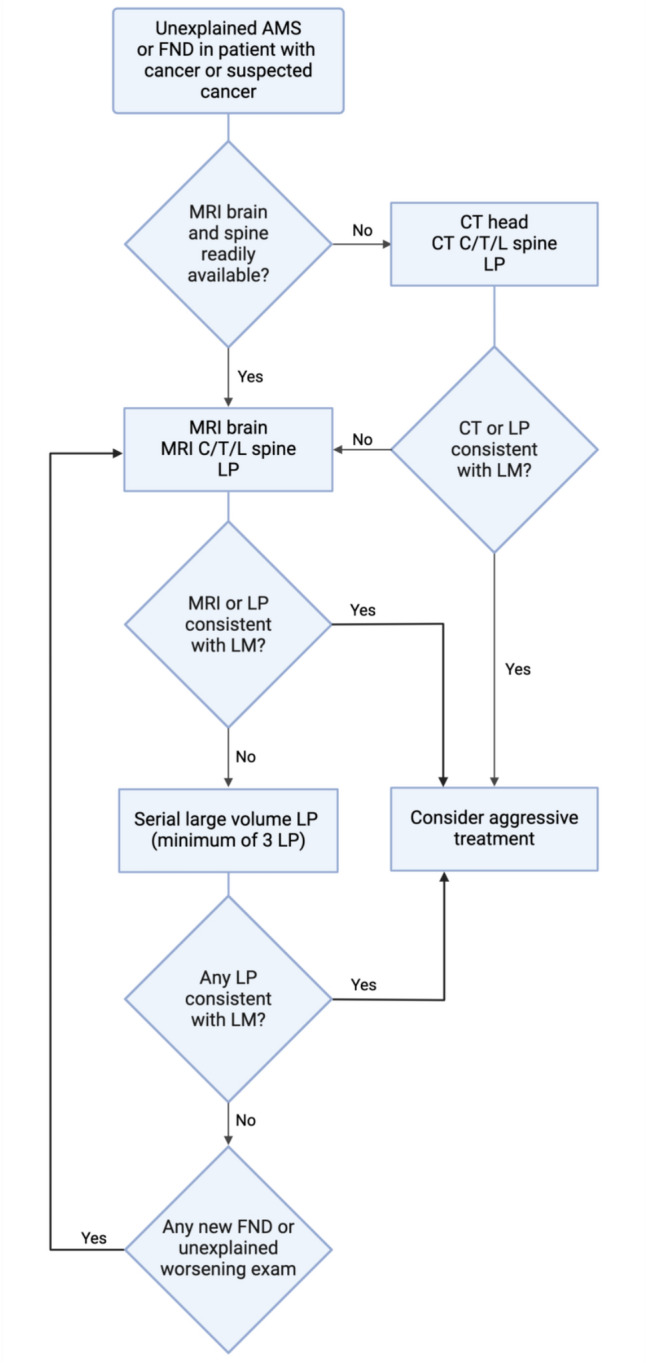
Approach to clinical signs suspicious for leptomeningeal disease This diagram describes the work flow to be followed in patients presenting with clinical signs and symptoms consistent with leptomeningeal disease (Table 2). It is to be used concordantly with infectious work-up and toxin screening. Because the presenting symptoms of LM are non-specific and the tests not highly-sensitive, this work flow should be employed in all patients with a cancer history or presenting with signs of undiagnosed cancer like rapid weight loss, or unexplained generalized fatigue. The work flow starts at the top rectangular box, diamonds denote decision points, and further rectangles denote results of those decisions. The diagram has no clear end point other than treatment because negative imaging or lumbar puncture results does not exclude LM. In patients in whom imaging and LP are not consistent with LM, clinical monitoring should be pursued while awaiting further clinical manifestation of disease.

Repeat MRI imaging should also be conducted in the setting of clinical progression without clear diagnosis and can increase test sensitivity. Initial lumbar punctures, if negative, should be repeated for the same reason [14, 25]. In the setting of high opening pressures, therapeutic large volume lumbar punctures are often performed. All available fluid should be sent for cytological analysis to improve test sensitivity. Figure 4 describes a work flow that can be followed in patients presenting with clinical signs and symptoms consistent with LM (Table 2). As the presenting symptoms of LM are non-specific and the tests not highly-sensitive, this work flow can be employed in any patient with a cancer history or presenting with signs of undiagnosed cancer like rapid weight loss, unexplained fever, or generalized fatigue.

Several mechanisms have been proposed to explain the development of LM including hematogenous seeding through venous plexi, arterial blood supply, or permeation of the blood–brain-barrier (BBB) [4, 26]. Tumor cells can spread through the Batson venous plexus, a valveless network of veins in the vertebral column which communicates directly with the venous circulation of the central nervous system (CNS) [4, 26]. This pathway is particularly relevant for cancers with a high predilection for spinal metastases. Another proposed mechanism is through arterial seeding via the choroid plexus. The choroid plexus has fenestrated endothelium which can be compromised during tumorigenesis. Cancer-promoting proteases such as matrix metalloproteinases (MMPs) degrade the extracellular matrix and endothelial tight junctions, enabling metastatic cells to seed into the subarachnoid space [27]. Iatrogenic spread, although less common, can occur following surgical procedures for brain masses, where manipulation of the CNS increases the risk of cancer cells being introduced into the leptomeninges [4, 26].

Given the potential hematogenous and CSF routes of disease dissemination, CSF circulating tumor cells (CTCs), circulating tumor DNA (ctDNA), CSF CEA levels and convolutional neural network (CNN) models are also being used to diagnose LM in gastric cancer, to identify LM-specific molecular features, and to quantify disease burden. CSF-derived ctDNA was shown to have greater diagnostic power in detecting genomic alteration than plasma-derived samples [28]. And its sensitivity and specificity (87.0% and 93.8% respectively) are comparable to that of CSF cytology (71.9% and 100%, respectively) [29]. Cytology was still preferred to rule disease in but CSF CTCs found disease that cytology missed [29]. Serum and CSF CEA levels are also suggestive of disease. CEA level were found to be reliably higher in the CSF of patients with LM than those without, in a variety of primary tumors [30]. A rise in CSF concentration of more than 2 – 3% of the serum values without an increase in CSF albumin concentrations was suggestive of LM as well [31]. In the reviewed cases, elevated CSF CEA was used to narrow the search for an unknown primary tumor[32], to aid in diagnosis of LM, and to monitor for disease progression after being high on initial testing [17, 18, 32]. Convolutional neural networks have also been shown to facilitate CSF cancer cell screening with a mean average precision of 95%, a level of precision similar to that of expert cytologists. This technology was additionally able to differentiate between lung, gastric, breast, and pancreatic cancer cells with a mean average precision of almost 80%, yielding 10% more precision and three times the speed of experts [33]. A thorough attempt at diagnosis is critical prior to offering interventions for LM due to highly toxic side effects.

Due to the current rarity of LM, standardized and successful treatments elude us. Treatments range from purely palliative regimens including physical therapy and pain control to intravenous and intrathecal chemotherapy, immunotherapy, surgery, and brain and spine radiation. NCCN guidelines recommend systemic therapy specific to the primary cancer type and intrathecal chemotherapy with thiotepa, topotecan, etoposide, cytarabine, or methotrexate – doses and duration of treatment are not included [34]. In our review, there were nine cases where patients survived for five or more months, out of which five reported dosages and five reported durations of treatment. Treatments were consistent with previously recommended regimens in a 2010 Lancet Oncology review [35] in that methotrexate ranged from 10 – 12.5 mg in all cases except one where the patient received 5 mg per session. The reported intrathecal cytarabine doses, ranging from 20 – 40 mg, were on the lower end of the recommended 25 – 100 mg regimens [35]. The low prevalence of LM in gastric cancer has prevented any direct comparisons of intrathecal chemotherapy options, but a recent review of all LM reported significant improvement in time without symptoms or toxicity as well as time before neurological progression after intrathecal liposomal cytarabine when compared to methotrexate. Median overall survival was no different between the two groups [31]. In our study it is unclear whether the patients’ survival was a reflection of continuing to receive treatment or whether the continued treatment was a surrogate for continued good functional status and stage of disease at presentation. The Kaplan Meier curves do show an association between receiving systemic or intrathecal chemotherapy and increased survival. The fact that they do not demonstrate a change in survival with radiation may indicate that radiation has no survival benefit or that a clinician’s threshold to treat with radiation therapy in someone with poor performance status is lower – resulting in more patients with late-stage disease receiving radiation. These results suggest that using these treatments is reasonable in patients who are fit for treatment, and in whom continued treatment is consistent with their goals of care. It should also be noted that there was no consistent reporting of functional status at presentation or treatments received prior to presentation with LM; the decision to start treatment was often determined based on the severity of the patient’s presenting symptoms and their performance status which introduced selection bias as well.

The relative effectiveness of systemic versus intrathecal chemotherapy, or their combined use, remains unknown and warrants further study to determine the optimal treatment strategy in this challenging clinical scenario. The Kaplan Meier survival curve comparing treatment with or without systemic therapy in those who received intrathecal therapy showed no benefit in overall survival (Fig. 3d). There was a small difference in their median survivals but no statistically significant difference was found due to variation within each subgroup. This may be due to a true negative result or due to type II error in the setting of a small sample size. These results are additionally limited in that treatment regimens, severity of illness, and patient functional status likely differed between the groups. Platinum analogues such as oxaliplatin and cisplatin, which are first-line therapies in metastatic gastric cancer, demonstrate limited penetration of the central nervous system [36]. Systemic therapies incorporating variations of 5-FU, however, have shown the ability to cross the blood–brain barrier [37], offering a potential therapeutic avenue. In hematologic malignancies with CNS involvement, high-dose methotrexate or intrathecal methotrexate has been successfully utilized in combination regimens to address the limitations of monotherapy [38]. Extrapolating from these approaches, a combination of systemic and intrathecal chemotherapy may provide additional benefits in leptomeningeal gastric cancer, provided the patient can tolerate such an intensive combined regimen.

Identifying the specific mutational profile of LM and its response to treatment is a key challenge in controlling extent of disease. Tumor cells in the cerebrospinal fluid (CSF) are preserved from immune surveillance and attack [31], and frequent mutation breeds treatment resistance. The molecular landscape of LM can differ from its primary tumor [39]; for instance a patient with gastric cancer LM had CSF CTCs with nine identified mutations, four that were seen in the patient’s primary tumor, and five additional mutations in MDM2, TP53, KRAS, STK11, and ALK. These mutations were specific to the CTCs and not seen in other CSF cells [19]. Prakadan et al. also identified heterogeneity in cell mutations and the ability of the disease to create tumor subclones whose fractional abundance shifted over time in response to treatment [40]. In a study of CSF from patients with LM from any solid tumor, differences in copy number variation (CNV) also identified clusters of cells with similar gene expression. In the cases reviewed here, progression on one line of intrathecal chemotherapy prompted the addition of a secondary drug, most often cytarabine, but repeat molecular analysis with CSF fluid was never reported. This, along with new technologies that can characterize LM at the single cell level, may be of benefit. An evaluation using di-electrophoresis array (DEPArray) technology has been used for single cell sorting in CSF CTCs in a patient with LM secondary to HER2 positive gastric cancer; it found that 21.8% of CTCs were actually HER2 negative [41]. While this technology was not yet used at the molecular level, there is potential for its future use.

There is much need for the advancement of care in leptomeningeal carcinomatosis. In the absence of sensitive diagnostics, maintaining a high clinical suspicion for leptomeningeal disease is crucial, and diagnostics should be repeated in the absence of a concrete alternative diagnosis. In treatment, each patient’s goals and priorities are paramount. Survival from the time of presentation ranged from 3 days to 12 months, and continued monitoring with lumbar punctures and prolonged intrathecal treatment was reported in many of the longer surviving patients. The body of literate reviewed suggests that an aggressive approach with multiple modalities be considered. Negative cytology, the lack of symptoms, or the reduction of CSF tumor marker levels can be used as indicators of temporary disease response but should not be mistaken for cure. Finally, with the continued development of targeted therapies and technologies to better characterize leptomeningeal disease at a single cell level, interval CSF testing to identify changes in a specific cancer’s molecular landscape may better guide treatment regimens at disease progression.

## Abbreviations

AMS: Altered mental status
FND: Focal neurological deficits
MRI: Magnetic resonance imaging
CT: Computed tomography
C/T/L spine: Cervical thoracic and lumbar spine
LP: Lumbar puncture
LM: Leptomeningeal disease
